# Real‐world application of a fast stool DNA test for colorectal cancer screening in primary screening positive population

**DOI:** 10.1002/cam4.5521

**Published:** 2022-12-05

**Authors:** Yunfeng Zhu, Guodong Zhao, Weihua Ma, Xinmin Chen, Xiaofei Chen, Danning Li, Shuyan Zhao, Shangmin Xiong, Minxue Zheng

**Affiliations:** ^1^ Haining Hospital of Traditional Chinese Medicine Haining Cancer Prevention and Treatment Research Institute Haining Zhejiang China; ^2^ Zhejiang University Kunshan Biotechnology Laboratory Zhejiang University Kunshan Innovation Institute Kunshan Jiangsu China; ^3^ Suzhou VersaBio Technologies Co. Ltd. Kunshan Jiangsu China; ^4^ Suzhou Institute of Biomedical Engineering and Technology Chinese Academy of Sciences Suzhou Jiangsu China

**Keywords:** colorectal cancer, real word, screening, stool DNA test

## Abstract

**Background:**

Stool DNA test has been emerged as an effective noninvasive method for colorectal cancer (CRC) screening, but the real‐world performance of stool DNA test in Chinese population has rarely been reported.

**Methods:**

A total of 36,527 subjects were recruited in Haining City from January 2021 to December 2021. Participants underwent primary screening by taking both two‐samples fecal immunochemical tests (FITs) and high‐risk factor questionnaire (HRFQ), and those who tested either positive by FITs or evaluated to be high risk by HRFQ were recommended to undertake subsequent stool DNA test and colonoscopy.

**Results:**

Of 36,527 participants, 34,778 (95%) completed both HRFQ and FITs, 9947 (29%) showed positive results during primary screening, and the colonoscopy compliance rate was 49%. Of primary screening positives, 8733 (88%) completed stool sample collections, and colonoscopy results from 4293 eligible participants were used for analyzing the performance of stool DNA test. The sensitivities for detecting CRC and advanced adenomas (AA) were 100% (95% CI: 60–100%) and 40% (95% CI: 34–46%), and the area under curve (AUC) was 0.961 (95% CI:0.954–0.967) and 0.625 (95% CI: 0.609–0.641), respectively. The specificity of stool DNA test was 84% (95% CI: 82–85%). The false‐positive rate for stool DNA test is about 10% less than that of primary screening.

**Conclusion:**

Stool DNA test is a cost‐effective and promising alternative strategy for CRC screening in China.

## Graphical abstract

This study analyzed the performance of a new stool DNA test in a real‐world application for CRC screening in Chinese population, and indicated stool DNA test is a cost‐effective and promising alternative strategy for CRC screening in China.

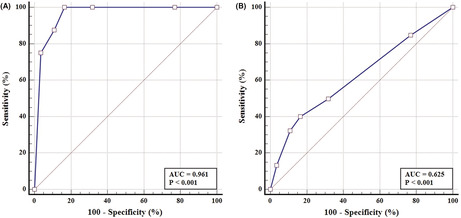

## 1 INTRODUCTION

Colorectal cancer (CRC) is a major cause of cancer burden in China, which caused about 408,000 new cancer cases and 195,600 deaths in the latest nationwide statistics for cancer incidence and mortality.^1^ Meanwhile, with fast economic development and the spread of western lifestyles in China,^2^ the rate of CRC incidence was significantly increased by 2.4% per year from 2000 to 2016.^1^ Early screening has been demonstrated to be an effective strategy to reduce the incidence and mortality of CRC.^3^ However, there are several challenges remaining in current practice of CRC screening in China, such as low compliance of colonoscopy, insufficient medical resources, low sensitivities of serum tumor markers, and financial burden of the government.^4^, ^5^ Therefore, a cost‐effective, accurate and noninvasive method is urgently needed to promote early CRC screening in China.

DNA methylation plays a key role in cancer proliferation, apoptosis, and differentiation,^6^ and thus, it has been emerged as a class of promising biomarkers for cancer diagnosis.^7^ In recent years, several DNA methylation‐based stool or plasma kits were developed and showed high sensitivities for advanced adenomas (AA) and CRC than those by traditional noninvasive methods.^8^, ^9^, ^10^ In 2014, Cologuard, the first stool multi‐target CRC screening test approved by FDA which included hemoglobin, *KRAS* mutations, and *BMP3* and *NDRG4* methylation sites as biomarkers, exhibited relatively high sensitivity and specificity.^11^ In 2016, a plasma‐based CRC screening test, Epi proColon 2.0 test, was approved by FDA, by utilizing a single *SEPT9* methylation site as the biomarker.^12^ Our research group has published a series of stool‐ or plasma‐based DNA methylation test for CRC early detection, including *SDC2*,^13^ *SFRP2*,^14^ *CLIP4*,^15^ *C9orf50*, and *KCNQ5*.^16^ Furthermore, we have developed a novel multiplex DNA methylation assay, ColoDefense, that could be applied to both stool and plasma samples.^17^, ^18^

Real‐world applications can reflect the true performance of a test in a target population, and support the regulatory and updates of screening guideline for new methods. Currently, most of DNA methylation tests are analyzed by “case–control” studies,^19^, ^20^ where samples incorporated are much “cleaner,” and thus may lead to overrated performance. For example, the sensitive and specificity of Epi proColon 2.0 test in case–control studies were about 75–81% and 96–99%,^21^ while those in a real‐world application study were only about 68% and 80%, respectively.^22^ However, no real‐world application data from the stool‐based DNA methylation tests used in China have been reported. In this study, we analyzed the performance of a simplified version of ColoDefense test in a real‐world application, to provide the evidence of stool DNA test as an alternative strategy for CRC screening in China.

## 2 METHODS AND MATERIALS

### 2.1 Study population

In this study, the population‐based CRC screening program was conducted in two villages (Yuanhua Village and Zhouwangmiao Village) of Haining City, Zhejiang Province, China, from January 2021 to December 2021. The inclusion criteria consisted of the following: residents with registered residence in the city aged 40–74; and the exclusion criteria were following: patients with CRC, severe heart, brain, lung disease, liver and kidney dysfunction, and severe mental disorder. According to the screening scheme, briefly, a three‐step screening process was adopted. Firstly, eligible individuals aged 40–74 years were invited to undertake two‐samples fecal immunochemical tests (FITs) and high‐risk factor questionnaire (HRFQ) by trained staff, and participants tested either positive by FITs or evaluated to be high risk by HRFQ were recommended to undertake subsequent stool DNA test and colonoscopy examination at designated hospitals. This study was performed according to the principles of the Helsinki Declaration and approved by the Institutional Review Boards of Haining Hospital of Traditional Chinese Medicine.

### 2.2 Primary screening

All participants underwent FITs and HRFQ at the same time, any one of which was high‐risk, then the subject was considered as positive during the primary screening. Written informed consent was obtained from all the study subjects before the FITs test begun. Two stool specimens from each participant at one‐week interval were used for FITs. Participants were instructed to collect about 10–50 mg stool samples from one bowel movement, place into the preservative buffer, and bring the samples back to the designated hospitals at room temperature in 24 h. FITs were tested by a One Step Fecal Occult Blood Test (colloidal gold) (Abon Biopharm, Hangzhou, Zhejiang, China), with the limit of detection for hemoglobin to be 100 ng Hb/ml. Subjects with at least one positive FITs were considered to be at high‐risk of CRC.

The HRFQ included the following risk index: (a) History of colorectal polyps; (b) History of familial adenomatous polyposis in first‐degree relatives; (c) Family history of CRC in first‐degree relatives (1 point for yes, 0 point for no); (d) Age (0 point for <55, 1 point for 55–64, 2 points for >65) (e) Gender (1 point for male, 0 point for female); (f) Smoking (0 point for nonsmokers, 1 point for smokers or quitters); and (g) Body mass index (0 point for <23, 1 point for ≥23). If the subject has any one of 1 or 2, he or she was regarded as high‐risk of CRC. The subject would be indicated as of high‐risk of CRC if the total score of 3–7 was ≥5, medium‐risk if that was 1–4, and low‐risk if that was 0.

### 2.3 Stool DNA test

Stool DNA test by using a multiplex real‐time quantitative polymerase chain reaction (qPCR) assay (ColoDefense2.0, Suzhou VersaBio Technologies Co., Ltd., Kunshan, China) detected methylated *SEPT9*, methylated *SDC2* and *ACTB* simultaneously in one PCR reaction. ColoDefense2.0 is a simplified version modified from ColoDefense 1.0^18^ by reducing 3 PCR replicates to 1 PCR replicate, and the reaction volume was increased from 30 μl to 50 μl. Written informed consent was obtained from all the study subjects before the stool sample collection and stool DNA test. The stool sample collection, stool DNA extraction, and bisulfite treatment were conducted according to previously published procedure,^18^ and the whole procedure last no longer than 6 h. A ColoDefense2.0 test included 25 μl template and 25 μl PCR master mix. ColoDefense2.0 were analyzed on LC480‐II thermal cycler (Roche Diagnostics, Basel, Switzerland) using the following cycling conditions: activation at 95°C for 30 min, 50 cycles of 95°C for 10 s, 56°C for 30 s, and final cooling to 40°C for 30 s. The stool sample was considered “invalid” if the *ACTB* Ct value was greater than 43.0. The Ct values of methylated *SEPT9* and methylated *SDC2* were put into a risk score calculation formula (Appendix S1). If the result was ≥3, the test was called “positive,” otherwise it was negative.

### 2.4 Colonoscopy and pathology

The high‐risk participants during primary screening were advised to undergo colonoscopy at designated hospitals. If the participant has not participated in colonoscopy, trained staff would continue to mobilize them up to 11 times. All colonoscopies were finished by trained physicians. Standard operation was followed for all colonoscopy examinations where endoscope reached cecum and the average withdrawal time was ≥6 min. Participants with abnormal colonoscopy results should have pathological diagnoses. An adenoma measuring ≥10 mm in size, with high‐grade dysplasia, or with ≥25% villous features was considered as AA, other adenomas were defined as nonadvanced adenoma (NAA). Cases other than CRC, AA, or NAA were considered as normal or other, where other group contained nonadenomatous polyps, hyperplastic polyps, diverticula, colitis, and other intestinal diseases.

### 2.5 Statistical analysis

All statistical analyses were performed with IBM SPSS (Windows Version 22.0). *Pearson* chi‐square test was used for comparison among different groups at a significance level of *p* < 0.05. The ROC curves were plotted by using the risk score of the ColoDefense2.0 test.

## 3 RESULTS

### 3.1 Study population characteristics

**FIGURE 1:**
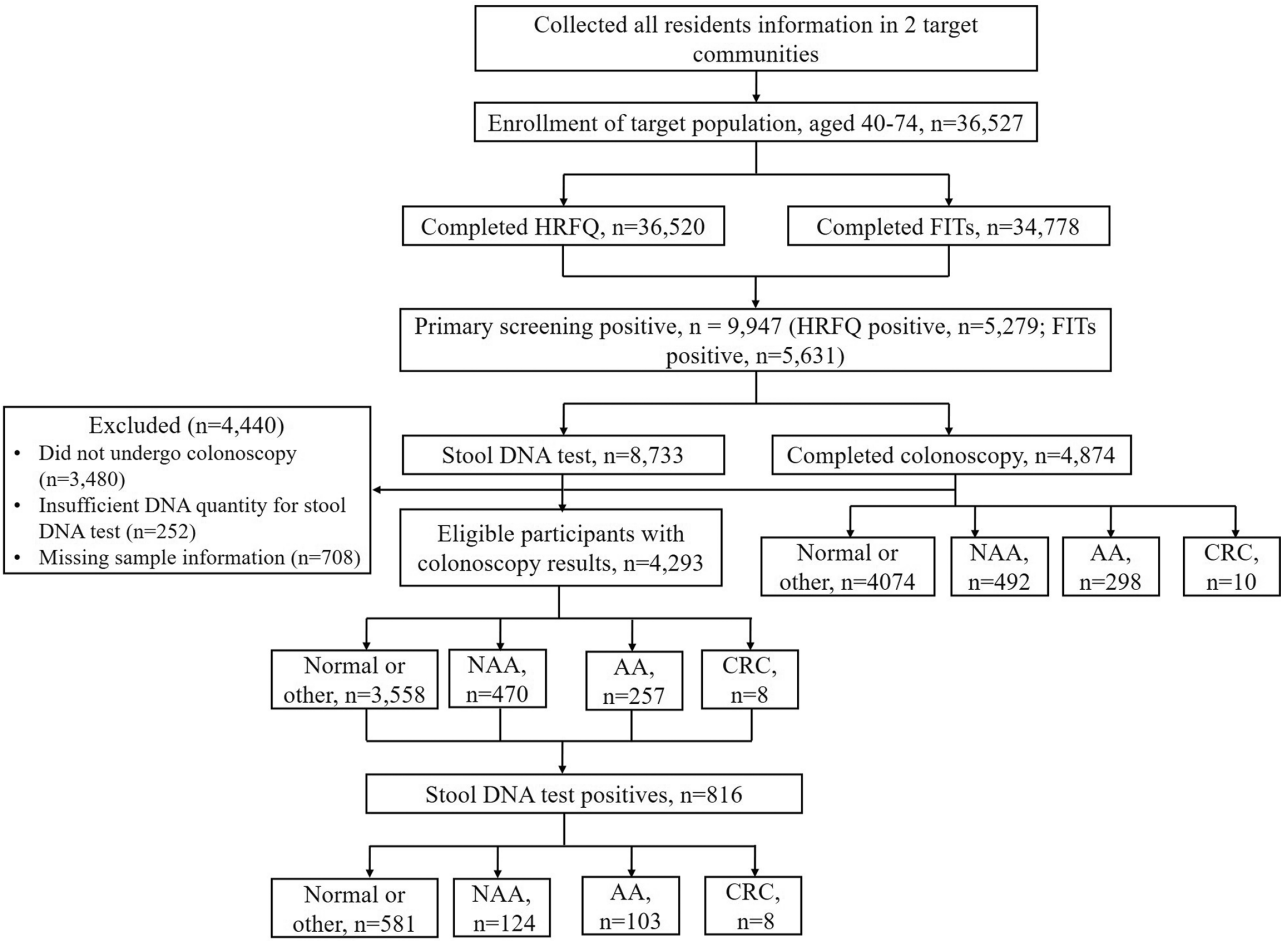
Flowchart of the study, including enrollment and outcomes.

A total of 36,527 subjects were recruited to undergo CRC primary screening, and 36,520 of them completed HRFQ and 34,778 completed FITs test (Figure 1). For details of invitees, 48% were males and 52% were females, about 15% of the participants were 40–49 years old, 38% were 50–59 years old, and 47% were 60–74 years old (Table 1). Regarding to the participants completed HRFQ and those completed FITs, 48% and 47% were males, respectively, and the age distribution at 40–49 years, 50–59 years, and 60–74 years were 15%, 38%, and 47%; and 13%, 38%, and 49%, respectively (Table 1).

**TABLE 1:** Characteristics of primary screening results.

	Total	Gender (*n* [%])		Age (*n* [%])		
		Male	Female	40–49	50–59	60–74
Eligible invitees	36,527	17,449 (48)	19,078 (52)	5372 (15)	13,810 (38)	17,345 (47)
Completed HRFQ	36,520	17,448 (48)	19,072 (52)	5370 (15)	13,807 (38)	17,343 (47)
Completed FITs	34,778	16,357 (47)	18,421 (53)	4667 (13)	13,217 (38)	16,894 (49)
Primary screening positives	9947	5604 (56)	4343 (44)	709 (7)	3221 (32)	6017 (60)
HRFQ positives	5410	3564 (66)	1846 (34)	277 (5)	1535 (28)	3598 (67)
FITs positives	5630	2823 (50)	2807 (50)	475 (8)	1948 (35)	3207 (57)
HRFQ and FITs positives	961	678 (71)	283 (29)	26 (3)	220 (23)	715 (74)
Completed colonoscopy	4874	2754 (57)	2120 (43)	249 (5)	1543 (32)	3082 (63)
CRC	10	5 (50)	5 (50)	1 (10)	2 (20)	7 (70)
AA	298	204 (68)	94 (32)	5 (2)	69 (23)	224 (75)
NAA	492	322 (65)	170 (35)	16 (3)	147 (30)	329 (67)
Normal or other	4074	2223 (55)	1851 (45)	227 (6)	1325 (33)	2522 (62)

Of 36,527 participants, 34,778 (95%) completed both HRFQ and FITs, and 9947 (29%) showed positive results during primary screening, 56% were males and 44% were females, only 7% were aged at 40–49, and 32% and 60% were aged at 50–59 and 60–74, respectively (Table 1). Among them, 5410 were only HRFQ positives and 5630 were only FITs positives, while only no more than 10% were co‐positives participants (961). We found the age distribution in only HRFQ or only FITs are similar to primary screening positives, while male in HRFQ positives were 10% more than that of primary screening, and male and female both took up 50% for FITs positives (Table 1).

### 3.2 Colonoscopy compliance rates and findings in the primary screening

**FIGURE 2:**
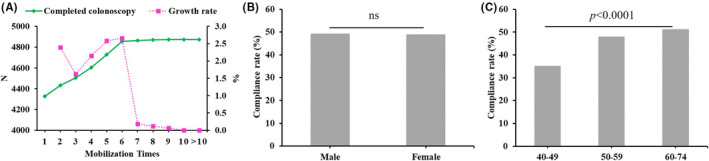
Colonoscopy compliance rate in this study. (A) Mobilization times of colonoscopy; (B) Gender; (C) Age. ns, not significant.

Participants having positive results in the primary screening were recommended for subsequent diagnostic colonoscopy. For 9947 positive cases, 49% (4874) completed colonoscopy and 800 colorectal neoplasms were found after pathological diagnoses. In order to increase participation in colonoscopy, we did more than 10 rounds of mobilization for those positive participants. For first round of mobilization, the colonoscopy compliance rate was 44%, and at second to sixth round, a small number of positive participants were willing to participate colonoscopy, and after the sixth round, the colonoscopy participation was barely increased (Figure 2A). For those who completed colonoscopy, the proportion of male and female seemed to have no significant difference, but the participation for different ages showed a significant difference (Figure 2B, C).

For 10 CRC cases found by colonoscopy, 100% of them were identified by FITs, and HRFQ only detected 30% CRC patients. While for AA, although FITs also detected more cases than HRFQ, the proportion of AA found by HRFQ was increased, and this phenomenon was more pronounced in NAA (Table 2). Similar to results during in the primary screening, the co‐positives cases also only accounted for less than 10% for those who completed colonoscopy.

**TABLE 2:** Findings in primary screening for FITs and HRFQ.

	CRC		AA		NAA		Normal or other
	*n* (%)	PPV (%)	*n* (%)	PPV (%)	*n* (%)	PPV (%)	*n* (%)
Colonoscopy (*n* = 4874)	10	0.2	298	6	492	10	4074
FITs positives (*n* = 2874)	10 (100)	0.3	222 (74)	8	287 (58)	10	2355 (58)
HRFQ positives (*n* = 2478)	3 (30)	0.1	112 (38)	5	252 (51)	10	2111 (52)
FITs and HRFQ positives (*n* = 478)	3 (30)	0.6	36 (12)	8	47 (10)	10	392 (10)

Abbreviation: PPV, Positive predictive value.

### 3.3 Characteristics of stool DNA test

The primary screening positive participants were also required to finish stool DNA test, and 8733 (88%) completed stool samples collection. Among them, 252 cases were with insufficient DNA quantity, and thus could not be processed for further stool DNA tests. Seven hundred and eight missed sample information and 3480 participants did not completed colonoscopy. Therefore, only 4293 cases were included for analyzing the performance of stool DNA test (Figure 1). The stool DNA test identified 8 of 8 participants with CRC, at a sensitivity of 100% (95% CI: 60–100%). Among 257 participants with AA and 470 participants with NAA, stool DNA test detected 103 (40%, 95% CI: 34–46%) AA and 124 (26%, 95% CI: 23–31%) NAA. Among the 3358 participants with normal or other results on colonoscopy, the specificity was 84% (95% CI: 82–85%) (Table 3).

**TABLE 3:** Sensitivity, specificity and PPV of the stool DNA test.

Finding	Colonoscopy (*n* = 4293)	Stool DNA test (*n* = 4293)			
	*n*	Positives results (*n*)	Sensitivity (%, 95% CI)	Specificity (%, 95% CI)	PPV (%)
CRC	8	8	100 (60–100)	—	1
AA	257	103	40 (34–46)	—	13
NAA	470	124	26 (23–31)	—	15
Normal or other	3558	581	—	84 (82–85)	—

**FIGURE 3:**
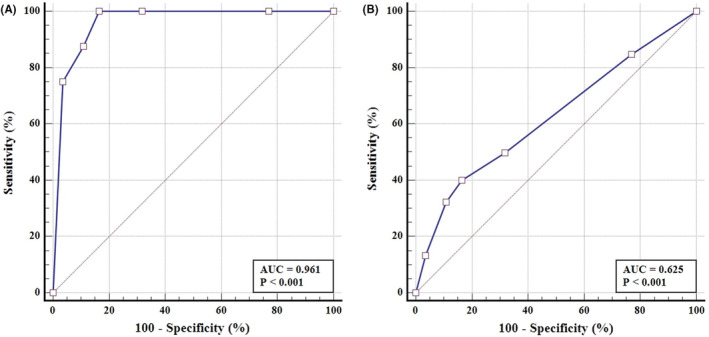
Receiver operating characteristic (ROC) curve of stool DNA test for detection of CRC (A) and AA (B).

**FIGURE 4:**
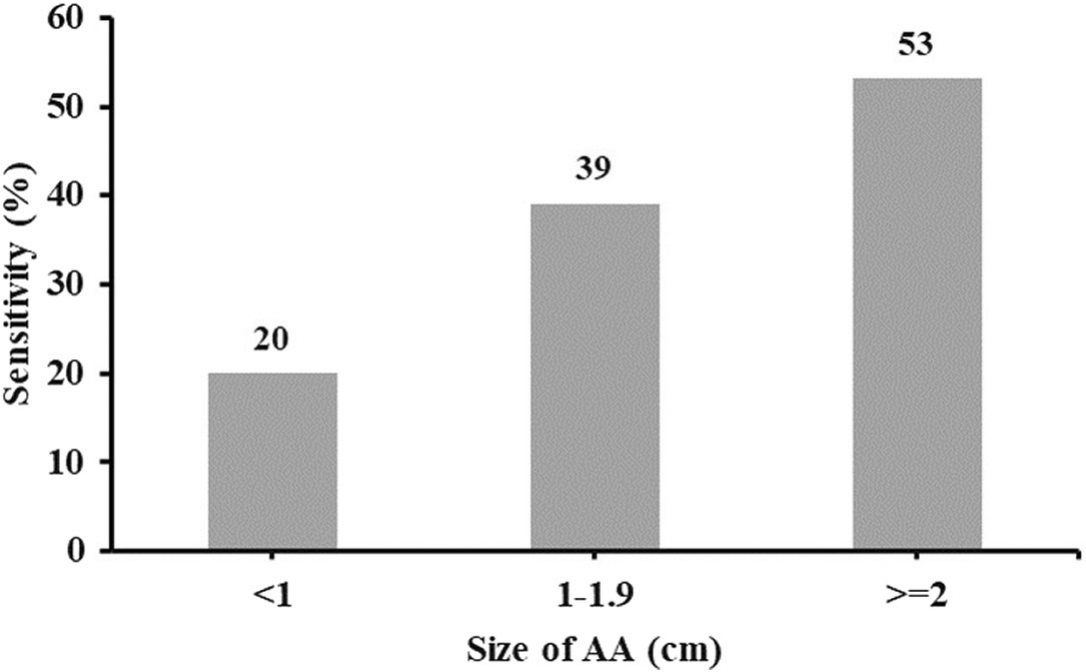
Sensitivity of stool DNA test for detection of different size of AA.

The AUC for stool DNA test showed an excellent performance on distinguishing CRC from normal or other cases with a result of 0.961 (95% CI:0.954–0.967), and the AUC of stool DNA test also displayed a significantly difference between AA and normal or other cases: 0.625 (95% CI: 0.609–0.641) (Figure 3). For AA less than 1 cm, the sensitivity was 20%, while for which size of 1–1.9 cm or larger than 2 cm, the sensitivities were increased to 39% and 53% (Figure 4).

### 3.4 Efficiency of stool DNA test

**FIGURE 5:**
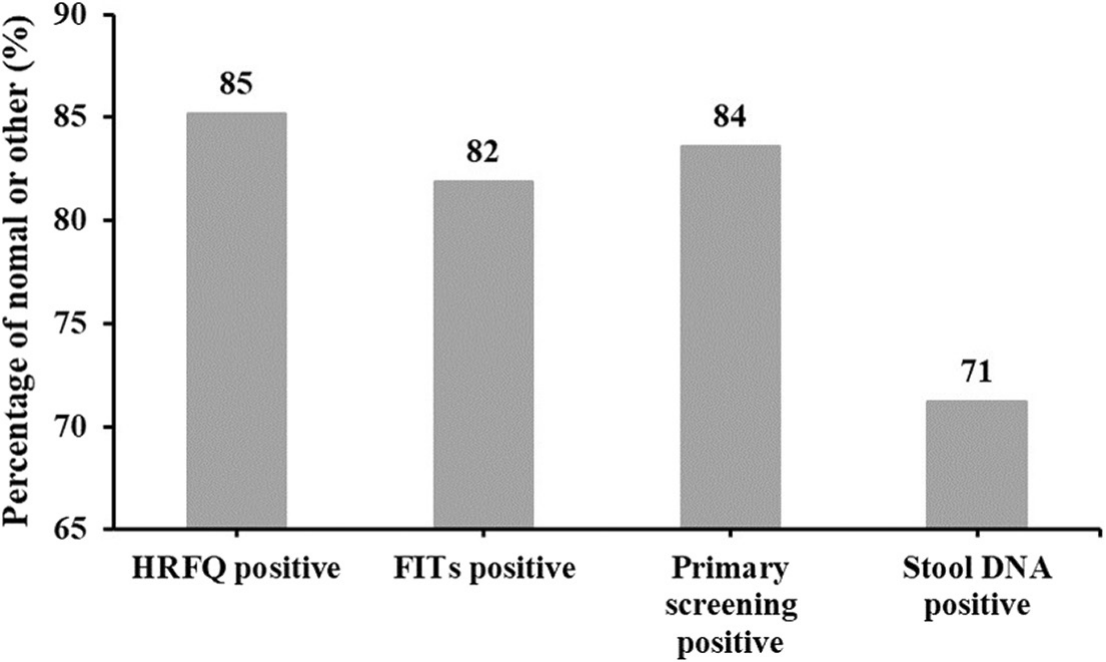
False positive results for different screening strategies.

By comparing the positive predictive values (PPVs) of HRFQ, FITs, primary screening, and stool DNA test, we found that stool DNA test exhibited the highest PPVs for CRC, AA, and NAA than all the other screening strategies (Table 2, Table 3), which indicated more normal or other participants were reported positive during primary screening (Figure 5). For stool DNA test, 19% (816 of 4293) participants were tested positive (Table 3), which was 10% less than that of primary screening. Based on such positive rate and colonoscopy compliance rate, if 36,527 subjects directly participated in stool DNA test rather than HRFQ and FITs, it would only result in about 6940 positive cases, which will reduce 1473 (3007 × 49%) cases to participate in colonoscopy, significantly reducing labor costs and medical costs. Meanwhile, if all the participants underwent colonoscopy after stool DNA test positive instead of primary screening positive, there would be only 816 cases that needed to go through the next step diagnosis, and no CRC cases would be missed compared to the current strategy.

## 4 DISCUSSION

CRC, as one of the most common malignant cancers in China, induced about 195,600 newly death cases in 2016.^1^ Early screening is the most important strategies for secondary prevention of CRC,^23^ and it has produced significant benefits in CRC prevention in the United States by promoting colonoscopy examination during the last 30 years.^3^ Haining, a city in East China, is one of the earliest cities in China to carry out CRC screening since 1978.^24^ Different from the United States, due to the financial burden for Chinese government and the low compliance of colonoscopy, several alternative primary screening strategies in Haining City were applied before colonoscopy examination, and HRFQ and FITs were among the most commonly used methods.^25^, ^26^ FIT is a common CRC screening strategy that has been recommended by guidelines in serval countries,^27^, ^28^ which showed relatively high sensitivity and specificity for CRC, but its sensitivities for AA and NAA were unsatisfactory.^29^ HRFQ is a widely used and cost‐effective CRC screening strategy by using a questionnaire to collection basic demographic information regarding risk factors, which showed good compliance, low cost and a relatively satisfactory performance during CRC screening, but its sensitivity for CRC was no more than 50%.^30^, ^31^ Therefore, HRFQ and FITs were often used in combination to make up for their respective deficiencies.^30^ In this study, HRFQ combined with FITs were also used for CRC primary screening, and the compliance rate of HRFQ was higher than that of FITs (~100% vs 95%). Combination of these two strategies significantly improved the positive detection of AA and NAA, but also induced about twice as many false positives compared with only one strategy. Meanwhile, the compliance rate of colonoscopy in this study was 49%, which was significantly higher than that in other regions of China,^32^ and such phenomenon indicated that the residents in Haining City have acquired high awareness of CRC screening due the long‐term routine CRC screening and promotion in the last 40 years.

In order to improve the performance of noninvasive methods for CRC screening, numerous new markers and technologies have been discovered and developed.^33^ Stool DNA test is a new method more sensitive than FITs for CRC and AA detection, and several commercial stool DNA test kits have been approved by Food and Drug Administration (FDA) or National Medical Products Administration (NMPA) and recommended for CRC screening in the updated guidelines in both United States and China.^11^, ^34^ For example, the first and only FDA‐approved stool DNA test, Cologuard, showed 92% and 42% sensitivities for CRC and AA with a specificity of 86% in a clinical trial including 9989 participants, and the comparison of FIT demonstrated Cologuard was about 20% more sensitive in CRC and AA.^11^ *Wang* et al. reported the performance of a NMPA approved single methylated gene (*SDC2*) based stool DNA test in 2020, whose results indicated such kit could detect 84% CRC and 42% AA with a specificity of 98%.^35^ In 2022, *Jin* et al. compared the performance of two NMPA approved multiple‐targets based stool DNA test with FITs in Chinese population. The results indicated the sensitivities of stool DNA test‐I, DNA test‐II, and FIT for CRC were 91%, 93%, and 81%, respectively, and the sensitivities of stool DNA test‐I, DNA test‐II, and FIT for AA were 35%, 42%, and 26% with specificities of 91%, 93%, and 97%, respectively.^36^ However, the performance of most of the above stool DNA tests, expect for Cologuard, were acquired by “case–control” or “prospective head‐to‐head comparative” studies rather than the performance in real world. The real‐world application would introduce more interference, which may display a worse performance when compared with case–control study, while much more instructive for clinical applications.

In this study, we analyzed the performance of a novel multiple‐targets based stool DNA test, ColoDefense2.0, in a real‐world population after primary screening by HRFQ and FITs. The results demonstrated that the sensitivities of ColoDefense2.0 for CRC and AA were comparable to those of Cologuard, especially the sensitivities of AA at different sizes showed the same trend and similar data between ColoDefense2.0 and Cologuard. The whole procedure of ColoDefense2.0, including stool DNA extraction, bisulfite treatment and qPCR reaction, took no longer than 6 h, which is significantly shorter than that of Cologuard. Furthermore, the simplified ColoDefense only need one replicate test, while Cologuard would need to perform detection of DNA methylation, mutations, and FIT at once. Therefore, ColoDefense2.0 has distinct advantages in time cost, reagent cost, and labor cost compared to Cologuard, based on the above conditions, the overall cost of ColoDefense2.0 is about $40 (approximately 300 RMB), which is about one sixteenth of that for Cologuard (about $649).^37^ Due to individuals for specificity analysis in this study were from CRC high‐risk population and those with any other gastrointestinal disease were not excluded, the specificity of ColoDefense2.0 was affected. Nonetheless, the specificity of ColoDefense2.0 remains consistent with real‐world data on Cologuard (84% vs 84–86%).^38^

Furthermore, we found the false‐positive rate detected by ColoDefense2.0 was less than that of current primary screening strategy in Haining City. If we use ColoDefense2.0 as the alternative primary screening method in future routine screening program or to finish a second round of screening for those CRC high‐risk population before colonoscopy examination, only those with ColoDefense2.0 positive participants would need to undergo colonoscopy. In that case, it will greatly save colonoscopy resources and further improve the efficiency of CRC screening in Haining City. In most of the current guidelines for CRC screening in United States or China, stool DNA test has been recommended as a new strategy for CRC primary screening, thus in developed city in China, like Haining, using stool DNA test as the primary screening stool for CRC is a feasible plan in the future. While in other underdeveloped areas in China, due to that the cost of stool DNA test is still slightly higher than that of HRFQ and FITs, using HRFQ and FITs as the primary screening stools followed by the stool DNA test in the second round of screening might be the best choice for CRC screening.

## 5 CONCLUSION

HRFQ and FITs are the most commonly used methods for CRC primary screening in China, and colonoscopy is the gold standard for CRC detection. In this study, we evaluated the performance of the stool DNA test in CRC primary screening positive population, and we found that the stool DNA test will emerge less false‐positive cases with higher sensitivity compared with HRFQ and FITs, and as a noninvasive diagnostic method, stool DNA test is more acceptable than colonoscopy. In conclusion, a novel and fast stool DNA test showed high sensitivities and acceptable specificity for CRC screening in the real word, which is a promising and cost‐effective alternative strategy for CRC early screening in China.

## AUTHOR CONTRIBUTIONS

**Yunfeng Zhu:** Conceptualization (equal); data curation (equal); methodology (equal); project administration (equal); resources (equal); writing – original draft (equal). **Guodong Zhao:** Conceptualization (equal); formal analysis (equal); funding acquisition (equal); methodology (equal); software (equal); writing – original draft (equal). **Weihua Ma:** Conceptualization (equal); methodology (equal); project administration (equal); resources (equal); validation (equal); writing – original draft (equal). **Xinmin Chen:** Methodology (equal); project administration (equal); resources (equal); validation (equal). **Xiaofei Chen:** Methodology (equal); project administration (equal); resources (equal); validation (equal). **Danning Li:** Formal analysis (equal); investigation (equal); project administration (equal); validation (equal). **Shuyan Zhao:** Data curation (equal); formal analysis (equal); investigation (equal); project administration (equal). **Shangmin Xiong:** Formal analysis (equal); funding acquisition (equal); writing – review and editing (equal). **Minxue Zheng:** Conceptualization (equal); supervision (equal); writing – review and editing (equal).

## CONFLICT OF INTEREST

The authors state no conflict of interest.

## ETHICS APPROVAL STATEMENT

This study was approved by the Institutional Review Board of the Haining Hospital of Traditional Chinese Medicine, and the informed consent was obtained from all participating patients and healthy control subjects, and the study was performed according to the Declaration of Helsinki principles.

## INFORMED CONSENT

Informed consent was obtained from all individuals included in this study.

## Supporting information


Appendix S1.

## Data Availability

The datasets used and/or analyzed for the current study are available from the corresponding author upon reasonable request.
